# Self-reported evaluation of competencies and attitudes by physicians-in-training before and after a single day legislative advocacy experience

**DOI:** 10.1186/1472-6920-12-47

**Published:** 2012-06-22

**Authors:** Kristin M Huntoon, Colin J McCluney, Elizabeth A Wiley, Christopher A Scannell, Richard Bruno, Matthew J Stull

**Affiliations:** 1 New York College of Osteopathic Medicine, Old Westbury, NY, USA; 2 University of Washington School of Medicine, Seattle, WA, USA; 3 American Medical Student Association, 45610 Woodland Rd, Suite 300, Sterling, VA, 20166, USA; 4 George Washington University School of Medicine, Washington, DC, USA; 5 University of Southern California, Los Angeles, CA, USA; 6 Oregon Health & Science University, Portland, OR, USA; 7 Department of Emergency Medicine, University of Cincinnati School of Medicine, Cincinnati, OH, USA

## Abstract

**Background:**

Advocacy is increasingly being recognized as a core element of medical professionalism and efforts are underway to incorporate advocacy training into graduate and undergraduate medical school curricula. While limited data exist to quantify physician attitudes toward advocacy, even less has been done to assess the knowledge, skills, and attitudes of future physicians. The purpose of this study was to assess students’ experiences and attitudes toward legislative advocacy, cutting out using a convience sample.

**Methods:**

A paper survey based on previously validated surveys was administered to a convenience sample of premedical and medical student participants attending a National Advocacy Day in Washington, DC, in March 2011, both before and after their advocacy experiences. Responses were anonymous and either categorical ( or ordinal, using a 5-point Likert scale. Data were analyzed statistically to evaluate demographics and compare changes in pre- and post-experience attitude and skills.

**Results:**

Data from 108 pre-advocacy and 50 post-advocacy surveys were analyzed yielding a response rate of 46.3%. Following a single advocacy experience, subjects felt they were more likely to contact their legislators about healthcare issues (p = 0.03), to meet in person with their legislators (p < 0.01), and to advocate for populations' health needs (p = 0.04). Participants endorsed an increased perception of the role of a physician advocate extending beyond individual patients (p = 0.03). Participants disagreed with the statement that their formal curricula adequately covered legislative healthcare advocacy. Additionally, respondents indicated that they plan to engage in legislative advocacy activities in the future (p < 0.01).

**Conclusions:**

A one-time practical advocacy experience has a positive influence on students’ knowledge, skills and attitudes towards legislative advocacy. Practical experience is an important method of furthering medical education in advocacy and further research is necessary to assess its impact in a broader population.

## Background

The role of advocacy training in medical education has generated significant controversy [1]. While some have questioned the potential politicization of medical schools and academic medical centers [2], advocacy has increasingly been recognized as a core element of medical professionalism [3], and efforts are underway to incorporate advocacy training into graduate and undergraduate medical school curricula [4-6]. Although there is no unified definition of physician advocacy [7], a growing number of specialty societies now recognize advocacy as a professional responsibility [8,9] and some require advocacy training and experience for trainees [10]. One compelling working definition of physician advocacy is “action by a physician to promote those social, economic, educational, and political changes that ameliorate the suffering and threats to human health and well-being that he or she identifies through his or her professional work and expertise.” [7] By virtue of the role physicians play in society and the unique features of the doctor-patient relationship [11,12], physicians and physicians-in-training are uniquely suited to advocate with and for patients.

To develop effective physician advocates, longitudinal skill development is essential from the outset of training [13]. The importance of this objective at the medical school level is outlined in the AAMC’s Medical School Objectives Project (MSOP) [14] and there are several medical schools with novel health policy programs [15-19]. These programs are highly valued by the limited number of trainees able to access them [20]. Exposure to policy, however, is not synonymous with exposure to advocacy, and most physicians-in-training do not have access to a comprehensive advocacy curriculum or even isolated advocacy experiences in formal curricula.

The aim of our study was to evaluate the impact of a single, half-day advocacy experience on a group of premedical and medical students participating in a National Advocacy Day as part of the American Medical Student Association’s (AMSA) Annual Convention in Washington, D.C. Participants were attendees at the Convention who elected to participate in Advocacy Day. Convention attendees are self-funded; there was no additional cost to participate in Advocacy Day. We evaluated the impact through pre- and post-surveys to assess knowledge, skills, and attitudes regarding legislative advocacy. While many organizations offer comparable advocacy opportunities on Capitol Hill for physicians and physicians-in-training, no previous studies have included a pre- and post evaluation of such an experience for American physicians-in-training. We sought to evaluate the impact of a single, practical advocacy experience on the attitudes and perspectives of physicians-in-training in the context of a growing recognition of the need for advocacy training.

## Methods

### Population

We surveyed a convenience sample of participants attending AMSA’s Advocacy Day in Washington, D.C., in March 2011. Participants chose a topic area with a specific legislative “ask” to discuss during visits with Congressional offices. Topic areas included healthcare access, student debt reduction, global health funding, and lesbian, gay, bisexual, and transgender (LGBT) health equity. As part of this event, physicians-in-training had the option of participating in a pre-Convention webinar and/or a two-hour in-person training session. Meetings were coordinated for participants with legislators and/or their staff. Each participant was scheduled to meet with a minimum of two federal Congressional offices in groups of two or more, and a concerted effort was made to ensure that physicians-in-training met with their respective legislators. Participation in the study was completely voluntary and no incentives were offered for participation. If participants consented, they were instructed to complete a pre-survey prior to training and a post-survey following the training and advocacy experience on Capitol Hill. Participants who did not participate in the in-person training did not receive surveys. Surveys were returned on-site or mailed directly to the AMSA national office.

### Survey design and testing

We designed the survey instrument to evaluate participants’ skills, attitudes, and past experiences with advocacy based on previously validated surveys in the existing literature [21,22]. Text was updated to align with the pre/post nature of the study. We used a paper survey to gather data from participants. Basic demographic information was collected. In addition, the survey and post-surveys each contained 14 identical five-point Likert scale questions. The post-survey contained an additional three five-point Likert scale questions. We designed these questions to collect medical students’ self-assessment of knowledge, skills and attitudes toward legislative advocacy. This study was conducted with Oregon Health & Science University Institutional Review Board approval.

### Data collection

In compliance with institutional review board requirements, we deidentified all data collected such that each survey response could not be linked to any specific individual. We numbered surveys to allow for comparison between individual pre- and post-advocacy responses.

### Measures and variables

Response categories were either categorical (gender, medical school, medical school class, political self-identification, etc.), or ordinal (strongly disagree, disagree, neutral, agree, or strongly agree). We included a single open-ended question to obtain qualitative feedback on the participants’ experiences. Measures were calculated percentages of collated responses per category based on the total number of completed responses.

### Statistical analysis

We calculated descriptive statistics for demographics and responses to survey questions. We performed paired t-tests to compare pre- and post-experience attitude and skill changes. The quantitative data were managed and analyzed using Microsoft Excel.

## Results

While precise numbers are not known, the American Medical Student Association estimates that approximately 200 students participated in the National Advocacy Day. Of these, approximately 150 students are believed to have participated in the pre-advocacy in-person training and would have received surveys. One hundred twenty-two participants completed the primary survey; of these, 14 were excluded, as they did not fully complete the survey. Of the remaining 108 participants, 50 participants completed a secondary survey, for a response rate of 46.3%. Fifty-eight percent of respondents were female, 40% were male and one participant did not respond. Pre-medical school trainees were 32% of respondents, pre-clinical trainees consisted of 40% of respondents (MSI - 16%, and MSII - 24%), clinical trainees consisted of 26% of respondents (MSIII - 18% and MSIV- 8%) and there was one M.D./Ph.D. student. The average age of respondents was 25.0 ± 4.2 years old (Table 1).

**Table 1:** Demographics of survey respondents

**RESPONSES**	**n (%)**
Primary Survey	122
Secondary Survey (response rate)	50 (46.3%)
GENDER^*^	**n (%)**
Female	29 (58.0%)
Male	20 (40.0%)
Other	1 (2.0%)
**AGE**	**25.0 ± 4.2 years**
**YEAR OF TRAINING**^**†**^	**n (%)**
Pre-medical school	16 (32.0%)
Medical school, pre-clinical	20 (40.0%)
MS1	8 (16.0%)
MS2	12 (24.0%)
Medical school, clinical	13 (26.0%)
MS3	9 (18.0%)
MS4	4 (8.0%)
M.D./Ph.D. training	1 (2.0%)
**TOPIC**^**‡**^	**n (%)**
Health Care for All	28 (56.0%)
Student Debt	10 (20.0%)
Global Health	7 (14.0%)
LGBT	5 (10.0%)
**PRIOR EXPERIENCE**^**§**^	**n (%)**
No prior experience	19 (38.0%)
At least one prior experience	31 (62.0%)

^*^Freeform response

^†^Participants responded to the prompt: “Year of Training (select one)”.

^‡^Participants responded to the prompt: “What topic will you be lobbying on (select one)?”.

^§^Participants responded to the prompt: “What type of contact have you had with a legislator or staffer prior to today’s visits (check all that apply)?” If the response was anything other than “None”, participants were said to have had at least one prior experience.

The majority (56%) of participants chose to advocate in support of improving health care access. A fifth of participants chose to advocate on student debt reform (20%). The remaining students were split with 14% choosing to advocate for global health funding and 10% advocating for LGBT health equity (Table 1).

We polled participants on their prior experience with in-person legislative advocacy. For more than a third (38%) of participants, this was their first legislative advocacy experience. The remainder indicated having engaged in at least one prior advocacy activity (Table 1). Nineteen participants (38%) had prior in-person advocacy experience.

For the remainder of the survey, participants self-assessed their knowledge, skills, and attitudes regarding legislative healthcare advocacy, both prior to and immediately following Congressional advocacy visits to assess any changes as a result of the Advocacy Day experience. We included three additional statements in the post-survey to assess preparedness and attitudes following the advocacy experience.

### Knowledge

As the survey was administered almost a year after passage of the Patient Protection and Affordable Care Act (PPACA), we assessed knowledge with the statement, “I understand the major provisions of the recently enacted health care reform legislation (Patient Protection and Affordable Care Act).” Although most of the participants felt they had a strong grasp on the content of PPACA prior to lobbying, a 0.3 Likert scale increase was noted in the post-advocacy survey (p = 0.03) (Table 2).

**Table 2:** Pre-and post-advocacy mean Likert score responses to survey statements

Survey Statement^*^	**Pre-score (mean)**	**Post-score (mean)**	**Difference Post-Pre**	**p-value**
**KNOWLEDGE**				
I understand the major provisions of the recently enacted health care reform legislation (Patient Protection and Affordable Care Act).	3.60	3.90	+0.30	0.03
My medical school curriculum has provided me with sufficient health legislative advocacy training.	2.02	1.99	−0.03	0.89
I can describe how public policy affects the health of populations that I serve.	3.60	4.07	+0.47	0.01
I can identify the opportunities available for physicians-in-training to function as health advocates.	3.24	3.82	+0.58	<0.001
**SKILL**				
I am able to effectively communicate my position to my legislators and/or their staffers.	3.42	3.99	+0.57	<0.001
I know what to expect when I meet with legislators and their staffers.	3.18	3.68	+0.50	<0.001
**ATTITUDE**				
As a physician-in-training, I believe I can influence policy.	4.16	4.20	+0.04	0.73
As a physician-in-training, I believe it is important to contact my legislators about issues important to my future patients.	4.50	4.64	+0.14	0.13
As a physician-in-training, I believe it is important to contact my legislators about issues that will affect the way I will practice medicine.	4.26	4.66	+0.40	0.03
Participating in the legislative process is a professional responsibility of physicians	3.80	4.04	+0.24	0.17
Meeting with legislators is a worthwhile use of my time.	3.74	4.40	+0.66	0.007
It is part of my role as a physician-in-training to advocate for populations' health needs within society.	4.10	4.54	+0.44	0.04
I feel that my role as a health advocate extends beyond the individual patient(s) I am treating.	4.24	4.70	+0.46	0.03
I plan to engage in health legislative advocacy activities in the future.	3.88	4.52	+0.64	0.006
**POST-SURVEY**^**†**^				
After participating in Advocacy Day, I feel more prepared to be a physician-advocate.	n/a	4.30	n/a	n/a
Participating in Advocacy Day has increased my commitment to engaging in health advocacy activities.	n/a	4.37	n/a	n/a
I am more likely to encourage others to engage in health advocacy activities as a result of my participation in Advocacy Day.	n/a	4.46	n/a	n/a

^*^Participants were asked to react to the following statements using a 5-point Likert scale with 1 being “Strongly disagree” and 5 being “Strongly agree”

^†^These statements were included only on the post-survey.

We surveyed participants on their baseline knowledge prior to any of the advocacy training provided by AMSA by prompting them with, “My medical school curriculum has provided me with sufficient health legislative advocacy training.” We obtained the lowest Likert scale scores of any prompts in the survey in response to this statement; the pre-survey average was 2.02 and in post-advocacy surveys this remained fairly constant at 1.99 (p = 0.89). In order to demonstrate an understanding of the role of policy upon patients, we asked participants to respond to the prompt, “I can describe how public policy affects the health of populations that I serve.” An average 0.26 Likert scale score increase was observed in post-advocacy respondents compared to their pre-survey responses (p = 0.02) (Table 2).

To determine whether knowledge of the legislative process would empower participants to partake in future opportunities, we prompted participants with, “I can identify the opportunities available for physicians-in-training to function as health advocates.” The post-survey responses showed an increase of 0.58 from pre-surveys, suggesting that from this single day experience, participants could identify ways in which to be active health advocates (p < 0.001) (Table 2).

### Skills

We used several prompts to gain insight into whether the Advocacy Day experience further developed participants’ legislative advocacy skills. The first prompt sought to evaluate their ability to make a legislative ask: “I am able to effectively communicate my position to my legislators and/or their staffers.” Post-survey data showed an increase of 0.57 on the Likert scale, indicating an increased confidence in participants’ ability to communicate (p < 0.01). To address whether the Advocacy Day experience helped participants craft goals and expectations for their meetings with Congressional leaders, we included the prompt, “I know what to expect when I meet with legislators and their staffers.” Post-survey measurements produced a 0.5 increase in Likert scale scores as compared to the pre-survey (p < 0.001) (Table 2).

The last skills-oriented category assessed participants’ attitudes toward visits and their role in political advocacy, both currently and in the future. Participants responded to the prompt, “As a physician-in-training, I believe I can influence policy.” We noted strong support for this statement (4.16 before and 4.20 after) but no statistically significant difference (p = 0.72) between pre-advocacy and post-advocacy responses. (Table 2).

### Attitude

We prompted participants, “As a physician-in-training, I believe it is important to contact my legislators about issues important to my future patients.” Participants strongly agreed with this statement, with a pre-advocacy survey average of 4.50 and a post-advocacy survey average of 4.64; the difference of 0.14 was not statistically significant (p = 0.13). Participants responded to the prompt, “As a physician-in-training, I believe it is important to contact my legislators about issues that will affect the way I will practice medicine.” The post-advocacy survey yielded a 0.4 increase in comparison to pre-advocacy survey results (p = 0.03) (Table 2).

To determine whether Advocacy Day participation would affect participants’ view of advocacy as a professional responsibility, we prompted respondents with, “Participating in the legislative process is a professional responsibility of physicians”. Post-advocacy responses showed a non-significant increase of 0.24 from pre-advocacy results (p = 0.17). To assess participants’ perception of the value of legislative advocacy, we prompted respondents with, “Meeting with legislators is a worthwhile use of my time.” This resulted in a substantial difference, with pre-advocacy surveys averaging 3.74 and post-survey responses yielding an average of 4.4 (p = 0.007) (Table 2).

**Figure 1:**
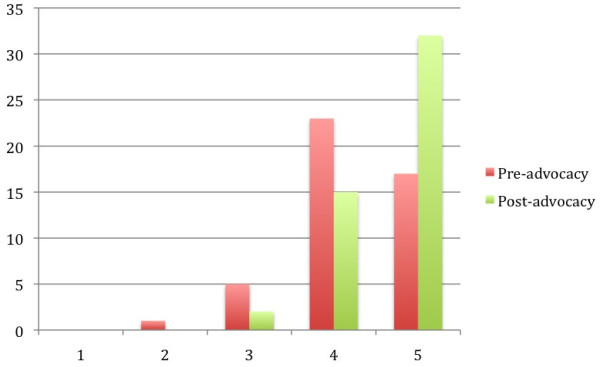
**Responses to “I plan to engage in health legislative activities in the future”.** Chart showing the number of responses received for each Likert score value (1 = Strongly Disagree to 5 = Strongly Agree) on pre- and post-advocacy surveys in response to the statement, “I plan to engage in health legislative activities in the future.” The mean Likert value increased from 3.88 to 4.52 (a change of 0.64) (p < 0.006) between pre- and post-advocacy surveys.

To address participants’ attitudes with respect to health inequities, we prompted them with, “It is part of my role as a physician-in-training to advocate for populations' health needs within society.” We noted a 0.44 increase in the post-survey answers from 4.10 to 4.54 (p = 0.04). We asked participants to respond to the prompt, “I feel that my role as a health advocate extends beyond the individual patient(s) I am treating.” Post-advocacy survey answers yielded a 0.46 increase from the pre-surveys (p = 0.03). Finally, attitudes about participants’ willingness and desire to participate in future advocacy efforts were evaluated by the prompt, “I plan to engage in health legislative advocacy activities in the future.” We recorded another substantial increase between pre- and post-survey responses in this category with an increase of 0.64 (p < 0.006) (Table 2, Figure 1).

We included three additional statements on the post-survey to assess for possible changes of perspective following the advocacy experience. All three questions ranked highly with an average Likert score between “Agree” and “Strongly Agree”. The statement, “After participating in Advocacy Day, I feel more prepared to be a physician-advocate,” garnered an average score of 4.30; the statement, “Participating in Advocacy Day has increased my commitment to engaging in health advocacy activities,” had an average score of 4.37; and the final statement, “I am more likely to encourage others to engage in health advocacy activities as a result of my participation in Advocacy Day,” received an average Likert score of 4.46 (Table 2).

## Discussion

We believe that this study is the first to assess the changes in skills and attitudes of U.S. medical trainees as a result of a single day legislative advocacy experience. Although various physician and medical student organizations offer advocacy experiences, the AMSA Advocacy Day experience was open to all levels of medical trainees and was less limited in scope, as participants were able to select a topic and specific ask to discuss with Congressional offices from a predetermined set of options (healthcare access, student debt reduction, global health funding, and LGBT health equity). Thus, students were able to select an issue that resonated with them and that was aligned with their individual ethical/moral convictions or motivations.

Participants rated their exposure to health policy in formal curricula poorly. This may be attributable to the disproportionate representation of premedical and preclinical students among participants (Table 2). Nonetheless, this finding suggests a further need for medical schools to integrate a comprehensive longitudinal health policy curriculum throughout training, including the development of skills in addition to a review of current policy. Interestingly, our results show that the advocacy day experience itself promoted health policy knowledge: following the brief training and advocacy experience, participants indicated an increased knowledge of both PPACA and how health policy can affect the populations they serve (Table 2).

Likewise, after engaging in the advocacy day experience, participants were able to identify future opportunities to serve as health advocates (Table 2). While our respondents indicated that their health policy curricula are lacking (Table 2), our results suggest that advocacy events or projects with a hands-on component can be a valuable component of enhancing policy education. Our survey participants also demonstrated that, in addition to training on the skills and techniques of advocacy, the actual experience of meeting with legislators to discuss policy and communicate positions is an important component of empowering physicians-in-training as future advocates - after the advocacy experience, respondents indicated they felt better able to anticipate the goals and expectations of such a meeting, and felt that they were more prepared to be a physician-advocate (Table 2).

Some of the highest Likert scale scores, both from the pre- and post-surveys were those in the attitudes section. Our results continue to support the notion that physicians-in-training view advocacy as an extension of their duty to their patients and their care. While participants initially acknowledged a responsibility to advocate with and for underserved populations, their legislative advocacy experiences reinforced that commitment. Although Likert scale scores reflecting a positive attitude xadvocacytowards advocacy approached the ceiling of the survey, we still found a statistically significant increase following the legislative meetings, indicating that participants believed that the experience was worth the effort. Despite a high baseline, students expressed a significant increase in their plans to engage in health legislative advocacy in the future (Figure 1). Notably, the high mean Likert scores for the three statements exclusive to the post-survey indicated that participants had an increased commitment and were likely to engage in advocacy efforts in the future (Table 2).

Optional freeform comments provided by participants anecdotally support many of the findings of this study. One participant reported that, “Today was a wonderful opportunity for me to expand my knowledge on health care reform acts currently being reviewed. I had a great experience overall at advocacy and I was inspired to continue advocating!” Another participant described how, “This experience increased my enthusiasm to share my opinion with the legislative community.” One participant described the experience as “amazing,” noting that, “It relates to our future what we do now, and through this visit you get the sense that you're not just going to be a physician but a part of the system that suffers, aides, changes, etc.” Lastly, after experiencing three legislative meetings, one student wrote simply, “The first one was jarring. The second one, exciting. The third one was empowering.”

Our findings represent a small, self-selected sample of physicians-in-training who have opted to participate voluntarily in an advocacy experience limiting the generalizability of our results. Although Advocacy Day was theoretically open to any physician-in-training, advertising for the event was targeted at AMSA members and Convention attendees. As a result, the study sample may not be reflective of the attitudes and opinions of the general premedical or medical student population. While this may affect Likert scores for any specific question, it is less likely that this would impact the differences between paired pre- and post- scores, helping to mitigate any effects of sampling bias in the findings. Some statements had results that approached the ceiling of the survey, making it unlikely that any significant change could be assessed following the advocacy experience. This effect was most notable in the “attitude” category; however, most statements in this category still showed statistically significant changes allowing for analysis and interpretation.

These limitations are common to most studies to date regarding advocacy, largely due to the nascent stage of advocacy education in medicine. As such, our study represents foundational work in the practice and study of healthcare advocacy. While much thought and discussion must continue around the integration of advocacy into medical curricula, we demonstrate that even single experiences can improve the perceived commitment trainees have to advocacy. Further validation of our work is encouraged as the long-term effects of single and longitudinal experiences with advocacy should be assessed. Such longitudinal studies regarding participants’ continued involvement in advocacy activities would be valuable in demonstrating the effectiveness of early advocacy training in producing long-term behavior change and potential patient benefit. Future studies would benefit from increased sample size and advocacy experiences targeted at a broader range of students, ideally in situations in which participation is not optional. Lastly, an evaluation of existing advocacy programs in medical education and their effectiveness would allow for the development of best practices and allow for further dissemination of methods for advancing these valuable skills.

## Conclusions

A single advocacy experience for physicians-in-training increases their appreciation of the importance of health care advocacy and of being active in the legislative process; and reinforces their perception that advocacy training and experiences are missing components of their schools’ curricula. Practical experience is an important method of furthering medical education in advocacy and encouraging this aspect of medical professionalism amongst future physicians.

## Competing interest

The authors have no conflicts of interest to disclose.

## Authors’ contributions

KMH participated in study design, data collection, and drafted the manuscript. CJM participated in data collection, data analysis, drafted the manuscript and made subsequent edits. EAW conceived of the study, participated in study design, data collection and helped to draft the manuscript. CAS participated in data collection, analyzed the data, and helped to draft the manuscript. RB participated in data collection, data analysis, and helped to draft the manuscript. MJS conceived of the study, participated in study design, data collection, and helped to draft the manuscript. All authors read and approved the final manuscript.

## Pre-publication history

The pre-publication history for this paper can be accessed here:

http://www.biomedcentral.com/1472-6920/12/47/prepub
